# Crucial and fragile: a multi-methods and multi-disciplinary study of cooperation in the aftermath of the COVID-19 pandemic

**DOI:** 10.3389/fpubh.2024.1368056

**Published:** 2024-05-16

**Authors:** Valentina Rotondi, Masiar Babazadeh, Giuseppe Landolfi, Marghertia Luciani, Laura Uccella, Samuele Dell'Oca, Michel Rosselli, Luca Botturi, Maria Caiata Zufferey

**Affiliations:** ^1^University of Applied Sciences and Arts of Southern Switzerland, Manno, Switzerland; ^2^Ente Ospedaliero Cantonale (EOC), Bellinzona, Switzerland

**Keywords:** interpersonal cooperation, COVID-19 pandemic, crisis management, collaborative decision-making, mixed methods

## Abstract

In addressing global pandemics, robust cooperation across nations, institutions, and individuals is paramount. However, navigating the complexities of individual versus collective interests, diverse group objectives, and varying societal norms and cultures makes fostering such cooperation challenging. This research delves deep into the dynamics of interpersonal cooperation during the COVID-19 pandemic in Canton Ticino, Switzerland, using an integrative approach that combines qualitative and experimental methodologies. Through a series of retrospective interviews and a lab-in-the-field experiment, we gained insights into the cooperation patterns of healthcare and manufacturing workers. Within healthcare, professionals grappled with escalating emergencies and deteriorating work conditions, resisting the “new normalcy” ushered in by the pandemic. Meanwhile, manufacturing workers adapted to the altered landscape, leveraging smart working strategies to carve out a fresh professional paradigm amidst novel challenges and opportunities. Across these contrasting narratives, the centrality of individual, institutional, and interpersonal factors in galvanizing cooperation was evident. Key drivers like established relational dynamics, mutual dependencies, and proactive leadership were particularly salient. Our experimental findings further reinforced some of these qualitative insights, underscoring the pivotal role of recognition and the detrimental effects of uncertainty on cooperative behaviors. While contextual and sample-related constraints exist, this study illuminates vital facets of cooperation during crises and lays the groundwork for future explorations into cooperative decision-making.

## 1 Introduction

Amid the profound challenges of global crises, cooperation crystallizes as the bedrock of human adaptability and collective resilience. To effectively counter these crises, cooperationis paramount, requiring the concerted efforts of nations (1, 2), institutions, corporations (3), and individuals (4). Although the vast benefits of cooperative endeavors during emergencies are evident, the personal sacrifices and the allure of prioritizing self-interest pose significant barriers (5).

Cooperation’s multifaceted nature means it’s influenced by numerous factors: the perennial tug-of-war between immediate self-interest and broader collective well-being (6); the challenge of harmonizing various group interests during emergencies (7); societal influences, including an innate preference to aid in-group members over out-group members (8); and overarching norms and cultural tenets (3, 9, 10).

The recent COVID-19 pandemic underscored the fragility and importance of cooperation among individuals (Bavel et al., 2020), revealing complex dynamics. In this light, our study delves deep into interpersonal cooperation within Canton Ticino, Switzerland. Employing a multi-method approach, our research synthesizes insights from a retrospective qualitative study with findings from a lab-in-the-field experiment conducted between April 2021 and November 2022, targeting healthcare and manufacturing workers.

Our qualitative research unearths varied dynamics of cooperation across healthcare and manufacturing sectors. Healthcare professionals grappled with myriad challenges, from surging workloads to fears of contagion. Their actions were predominantly reactive, aiming to mitigate the immediate repercussions of the pandemic. Conversely, the manufacturing sector showed adaptability, steering toward long-term operational adjustments and capitalizing on the unforeseen opportunities presented by smart working. Common threads like the pivotal role of recognition, especially in high-stress scenarios, were observed across both sectors. This insight framed our lab-in-the-field experiment, which quantitatively validated the impact of recognition on cooperative behaviors. The results spotlighted the ebbing of cooperation under persistent stress without a clear endpoint in sight.

Our research offers an intricate examination of cooperation during the COVID-19 pandemic in Canton Ticino, Switzerland. Through this meld of qualitative and quantitative methodologies, we derive a nuanced understanding of cooperation’s multifaceted nature. These findings have far-reaching implications, highlighting the need for astute strategies to nurture cooperation during crises.

For ease of navigation, this paper unfolds as follows: Section 1 reviews the literature on interpersonal cooperation. Section 2 establishes the context of our study in Canton Ticino. Section 3 outlines our methodological design, while Section 4 presents our findings. Finally, Section 5 provides a synthesis of our insights, reflecting on the broader implications and suggesting directions for future research and policymaking.

## 2 Literature review

### 2.1 Fostering interpersonal cooperation in the COVID-19 pandemic: challenges and opportunities for global public goods

Samuelson’s definition of public goods identifies two characteristics: non-rivalry in consumption and non-excludability (11). Non-rivalry means a public good’s use does not reduce its availability to others, while non-excludability indicates nobody can be excluded from its benefits, irrespective of their contribution. Global health, transcending borders, embodies a global public good (12), reflecting the interconnectedness of nations in addressing health challenges (1).

The COVID-19 pandemic underscores the significance of global public goods. It reveals strong incentives for free-riding when individual contributions appear insignificant. Inadequate cooperation can compromise public good provision, evident in the vaccine nationalism seen in the vaccine distribution (13). Marginalized populations face repercussions due to this disparity (14).

Our paper investigates interpersonal cooperation during crises, examining diverse individuals confronting a shared challenge. Cooperation’s essence lies in its dynamics, with humans oscillating between cooperation and defection. Cooperators bear costs for the group’s well-being [(5): 1560], while defectors pursue personal gain without considering collective interests. Despite its inherent costs, cooperation is pervasive, extending to interactions among unrelated individuals (4, 15), signifying its critical role in societies.

### 2.2 Factors affecting interpersonal cooperation

Various factors, including social norms, cultural influences, reciprocity, reputational concerns, and evolutionary mechanisms, influence the emergence and maintenance of cooperative behaviors among individuals. Additionally, the characteristics, rules, and dynamics of the group (or organization) to which individuals belong play a crucial role in fostering cooperation.

#### 2.2.1 Group-related factors

Group-related factors include elements like group membership, cohesion, and uniformity (16). Recognizing a common goal, feeling equal, satisfaction within the group, acknowledging interdependence, and shared identity all heighten interpersonal cooperation (17). The group’s organizational structure influences cooperation. Effective communication, clarity in roles, available tools and resources, and recognized leadership all impact cooperative behavior (18, 19). Leaders foster trust and a “we are all in this together” belief (20), facilitating coordination against external threats (16). Furthermore, seeing others as cooperative boosts one’s propensity to cooperate (21).

#### 2.2.2 Individual-level factors

Individual-level factors also influence cooperative behavior during unexpected and unknown events. Among these factors, trust in institutions, governments, and scientists plays a significant role (22). Trust in science is particularly crucial during epidemics, as it helps prevent small-scale outbreaks from escalating into large-scale emergencies. Trust in science, experts, and institutions determines citizens’ compliance with public health policies, restrictions, and guidelines (23). However, building and maintaining trust can be challenging in times of uncertainty and risk (24). The perception and handling of risk also play a crucial role in cooperation during crises. How individuals represent and interpret risk impacts their motivation to take action. When risk is viewed as a stimulating challenge, individuals are more motivated to act. Conversely, perceiving risk as a threat to be avoided diminishes individual motivation (25).

In interpersonal cooperation, particularly in the workplace, professional identity emerges as a critical aspect. Professional identity encompasses professional ethics, including ethical knowledge, beliefs, skills, and implicit and explicit norms related to one’s profession (26). Professional identity may conflict with institutional, family, or personal expectations, leading to conflicts at the intra-, interpersonal, and intergroup levels. Effectively managing these conflicts becomes essential for cooperation, as unresolved conflicts can lead to group dissolution while reinforcing interpersonal cohesion and cooperation dynamics.

#### 2.2.3 External factors

External levers can also be employed to enhance cooperation. Extensive research indicates that several mechanisms can effectively promote cooperative behavior. These include punishments (27), rewards (28), observability (29), and moral suasion (30).

Punishments can serve as a powerful tool to deter defection and promote cooperation. By imposing penalties or sanctions on individuals who engage in non-cooperative behavior, the costs of defection are heightened, thus incentivizing individuals to choose cooperative actions. This helps maintain social order and discourage free-riding tendencies (27). Conversely, rewards can act as positive reinforcements for cooperative behavior. When individuals are offered incentives or benefits for engaging in cooperative actions, they are more likely to contribute to the common good willingly. The prospect of receiving rewards can motivate individuals to prioritize collective interests over personal gains, fostering a culture of cooperation (28).

Observability, or the degree to which individual actions are visible or known to others, can significantly impact cooperative behavior. When people know their actions are being observed and evaluated by others, they tend to exhibit higher levels of cooperation. Social scrutiny creates pressure to conform to cooperative norms as individuals strive to maintain a positive reputation and avoid reputational costs associated with non-cooperative behavior (29).

Moral suasion involves appeals to individuals’ moral values and sense of ethical responsibility to encourage cooperative behavior. By emphasizing the moral dimensions of cooperation and highlighting its importance for the well-being of the group or society, individuals are more likely to engage in cooperative actions driven by their intrinsic motivation to do what is morally right (30).

### 2.3 Enhancing understanding of cooperation during crises: a mixed-methods study in real-world settings

Our paper builds upon the existing literature on cooperation during global crises, and it introduces several innovative elements that enhance our understanding of this phenomenon. Firstly, we adopt a mixed methods approach by combining a retrospective qualitative study with a lab-in-the-field experiment. This comprehensive approach allows us to capture the nuances of cooperation experiences during the COVID-19 pandemic and complement them with behavioral observations in a Public Goods Game, a well-established experimental tool in the social sciences for studying cooperation.

Secondly, we go beyond the conventional behavioral economics approach, which often relies on laboratory experiments with a sample of students. Instead, we investigate cooperation in real-world settings, specifically focusing on the healthcare and manufacturing sectors within Canton Ticino, Switzerland. By studying cooperation in the field, we are able to observe and analyze behaviors in the actual context where cooperation takes place, providing valuable insights that may not be fully captured in controlled laboratory environments.

Thirdly, our study examines both individual-level and contextual factors and explores their interplay in shaping cooperation. We recognize that cooperation is influenced not only by individual characteristics and motivations but also by the broader social and organizational contexts in which it occurs. By considering both individual and contextual factors, we offer a more comprehensive understanding of the complex dynamics that underlie cooperation during a crisis.

## 3 The context

Canton Ticino, in southern Switzerland, predominantly speaks Italian. With a diverse population and economy, it’s influenced by both Swiss and Italian traditions. Like many regions, it faced challenges during the COVID-19 pandemic, especially being close to Lombardy, a major pandemic hotspot in Europe. To control the virus’s spread, Canton Ticino enforced lockdowns and other measures.

During the pandemic, the healthcare sector had to restructure extensively. Hospitals like “Ospedale La Carità” and “Clinica Luganese” became dedicated COVID-19 centers. This transformation caused significant adjustments for healthcare workers, with many redeployed to new roles. On March 13, 2020, due to the escalating situation, the Swiss government even suspended the maximum workday length regulations for hospital staff.

Amidst these challenges, healthcare workers, especially those in COVID wards, faced immense pressure and moral dilemmas, as cited by (31). Cross-border health workers, making up 14% of the healthcare workforce in Ticino, dealt with the contrasting COVID-19 management strategies between Ticino and Northern Italy, fostering confusion. When Northern Italy became a pandemic epicenter on February 21, 2020, Italian authorities enforced stricter measures (32). In contrast, Ticino adopted a milder approach, emphasizing individual responsibility.

Consequently, these contrasting measures created uncertainty, especially for cross-border healthcare professionals regularly moving between the two regions. This emphasized the need for cooperation among healthcare workers.

The manufacturing sector too encountered challenges. Many companies in Ticino halted or reduced production due to supply chain disruptions, resulting in significant revenue losses. Reorganization required workers to adjust, underlining the importance of cooperation, which, despite uncertainties, showed resilience as the pandemic evolved. This study focuses on understanding this resilience amidst challenges faced by workers in health and manufacturing sectors.

## 4 Data and methods

The paper presents two consecutive studies on interpersonal cooperation during the Covid-19 pandemic. The first study employed a qualitative approach, referencing existing literature to grasp cooperation dynamics in this unique context. Through online interviews, researchers examined interpersonal cooperation’s intricacies, challenges, and influencing factors during the pandemic.

Using insights from the qualitative study, the next phase implemented a lab-in-the-field experiment. This aimed to further probe hypotheses regarding interpersonal cooperation under stress. By manipulating variables and observing behavior, researchers gleaned insights complementing the qualitative data.

Integrating qualitative and experimental methods facilitated a holistic understanding of cooperation during the pandemic. The qualitative study provided deep insights into real-life experiences, while the experimental phase enabled hypothesis testing. Together, they sought to enrich the understanding of cooperation in crisis contexts.

The Cantonal Ethical Committee reviewed both protocols. The qualitative study did not require ethical approval under Swiss human research law, but still followed the Declaration of Helsinki with participants giving oral consent. However, the experimental study received approval from the Ethical Committee before data collection (no. 2021-01914CE 3952).

### 4.1 Data

#### 4.1.1 Qualitative study

We conducted a qualitative study using Braun and Clarke’s methodology (33) for its depth. Through online interviews, we focused on healthcare workers from the Canton Hospital Organization (Ente Ospedaliero Cantonale, EOC) from April to December 2021. This timeframe captures Switzerland’s first three COVID-19 waves and the start of the vaccine roll-out in January 2021.

Using intensity and maximum variation sampling (34), we captured diverse cooperation experiences. With an EOC-affiliated physician, we identified key departments: intensive care, emergency, and internal medicine. Our sample aimed for diversity, factoring in gender, residence status, and deployment during the pandemic, duties, role, leadership, and age.

We invited potential participants; 45 showed interest, but 29 were finalized due to accessibility issues.

Simultaneously, we explored the manufacturing sector between April and May 2021 to compare findings with healthcare. Partnering with four local industries (fashion, furniture, electronics), we used a similar recruitment approach, resulting in a varied sample of 20 managers and employees from Ticino.

Overall, our diverse sample comprised 49 participants, detailed in Table 1.

**Table 1:** Sample of the qualitative study.

Healthcare workers (*n* = 29)
14 women, 15 men
8 cross-borders, 21 residents
19 working in COVID hospitals, 10 in non-COVID hospitals
10 working in emergency department, 14 in intensive care, 5 in internal medicine
8 physicians, 21 nurses
7 (out of 21) nurses and 8 (out of 8) physicians with professional responsibility
24–65 y.o., mean age 49 y.o.
Manufacturer workers (*n* = 20)
8 women, 12 men
12 crossborders, 8 residents
11 working in smartworking, 9 in presence
28–63 y.o., mean age 45[MOU2]

The entire research team discussed the results of the qualitative study in the healthcare and manufacturing sectors to identify operational hypotheses regarding the drivers and barriers of interpersonal cooperation that could be effectively tested in the experimental study. The hypotheses were pre-registered.

#### 4.1.2 Lab-in-the-field experiment

The results of the qualitative study were tested in a second experimental study. In this case, we investigated the propensity for cooperation among healthcare and manufacturing workers using a public good game (PGG). The PGG is a standard game in experimental social sciences (35). In the basic game, participants are given a small initial sum of money and must decide how much to contribute to a “common pool.” The resources transferred to the common pool are multiplied by a constant and then divided among the game participants. Each participant keeps for themselves the resources they did not contribute to the common pool. From a purely theoretical standpoint, according to Nash equilibrium, no participant should contribute anything to the common pool because any rational agent would maximize their own profit by keeping all the money for themselves, regardless of what others do. However, experimental literature shows that Nash equilibrium is rarely achieved. Typically (36), those who contribute more to the common pool are referred to as “cooperators,” while those who contribute less are called “defectors.”

The Public Good Game (PGG) was conducted using a between-subjects experimental design, incorporating four main treatment manipulations. In the baseline condition, participants engaged in the classic PGG for 10 rounds. In the recognition treatment, the top contributor was visually acknowledged with the display of two applauding hands on the screen. In the stress treatment, participants were required to make their decisions within a specific time limit indicated by a timer on the screen. In the extra-rounds treatment, participants unexpectedly received information that they had to play an additional 10 rounds. Additionally, three cross-treatments were introduced, where two treatments were combined. These cross-treatments resulted in the following three additional conditions: extra-rounds/stress, extra-rounds/recognition, and stress/recognition.

Consistent with common practices in experimental economics games, participants were remunerated based on their choices. On average, participants received approximately 22 Swiss francs per person, which were delivered through Amazon vouchers of equivalent value. The experiments lasted on average 45 min including the wearing of the sensors used to track their heart rate and electrocardiogram. After completing the experimental game, participants were administered a brief questionnaire. The questionnaire collected demographic information, employment details and included inquiries about psychological traits such as trust level and the Italian version of the Big Five Personality Traits, as proposed by Chiorri et al. (37). Additionally, the questionnaire encompassed two questions related to caution when interacting with people, a self-assessment of risk preferences, and people’s willingness to help others.

Furthermore, we employed the Global Preferences Survey (38) to measure pure generosity and positive reciprocity. Additionally, we incorporated a set of questions on cooperation, which had been validated by Lu et al. (39).

It should be noted that the original plan for the experiment was to be conducted online without any strategic interaction. However, due to the favorable epidemiological situation and the development of a dedicated computer platform for conducting on-the-move behavioral economics experiments, the decision was made to conduct the experiments in person with strategic interaction. This change in approach came with a trade-off: due to time and sample constraints, we were unable to include in the experiment an in-group/out-group manipulation which were originally included in the pre-registration.

Between May and November 2022, a series of experiments were conducted using a newly developed computer platform. The platform consists of multiple components, with one of its key features being the management of historical data from wearable sensors. It also includes functionalities for anonymized operator management and efficient handling of the experiment results. Moreover, the platform incorporates functional modules that offer additional features, such as the behavioral economics games, specifically the Public Good Game utilized in this study, and artificial intelligence modules that provide valuable insights beyond manual analysis capabilities. Through the utilization of this platform, participants’ heart rate and electrocardiogram were continuously monitored throughout the experimental sessions. The final working sample comprised 31 participants, resulting in a total of 767 observations.

### 4.2 Methods

#### 4.2.1 Qualitative study

Participants partook in semi-structured online interviews, recorded and subsequently transcribed verbatim. Ensuring confidentiality, all identifying details were removed. Adopting Braun and Clarke’s (33) thematic analysis approach, these transcripts were meticulously analyzed. To adhere to COVID-19 safety protocols, interviews were conducted online, incorporating strategies to enrich data quality as recommended by Caiata Zufferey and Aceti (40).

Each interview, informed by a semi-structured guide featuring open-ended prompts (e.g., “Reflect upon workplace conflicts during the pandemic: can you elucidate their origins and your response?”), was a dialog. Beyond the guiding questions, participants were free to explore and emphasize any topic they deemed relevant. Interviews varied in length, ranging from 40 to 80 min.

For a systematic thematic analysis, we embraced Braun and Clarke’s (33) iterative stages, commencing with an intimate understanding of the data, which involved revisiting the transcripts multiple times. This was followed by coding, theme identification, theme review, understanding inter-theme relationships, and finally, synthesizing findings for the report. A single researcher, who was also the interviewer, embarked on an inductive coding process, marking both manifest and latent content. Throughout the analysis, the research team convened regularly, deliberating on code assignments and interpretations. Any differences in perspectives were debated and resolved collaboratively, ensuring a multi-faceted understanding of the data.

#### 4.2.2 Lab-in-the-field experiment

In our analysis of data derived from lab-in-the-field experiments, we implemented a structured three-step statistical approach to elucidate the dynamics of interpersonal cooperation and the impact of various treatments.

##### 4.2.2.1 Step 1: initial comparative analysis

Initially, we employed a combination of non-parametric and parametric statistical tests to rigorously evaluate the data. Specifically, we utilized *T*-tests (a parametric test) and Mann–Whitney tests (a non-parametric test) for this purpose.

##### 4.2.2.2 Step 2: identifying predictors of cooperation

Subsequently, we harnessed the power of machine learning through the implementation of a Random Forest algorithm, utilizing data from questionnaires and wearable sensors. This advanced analytical method was selected for its proficiency in handling high-dimensional data and its ability to identify the most relevant predictors from a potentially large pool of characteristics. The algorithm analyzed a wide array of variables, including demographic information, psychological traits (such as trust and the Big Five Personality Traits), risk preferences, and measures of generosity and reciprocity, among others. The objective was to pinpoint specific attributes of participants that significantly influenced their propensity toward interpersonal cooperation.

##### 4.2.2.3 Step 3: exploring the influence of individual characteristics

In the final stage of our analysis, we applied a linear regression model to delve deeper into the relationship between individual contributions in the Public Good Game (PGG) and a set of explanatory variables. This encompassed treatment variables, the most salient predictors identified by the Random Forest, and additional control variables (e.g., gender, age, healthcare worker status). Linear regression was chosen for its effectiveness in quantifying the strength and direction of associations between the dependent variable (PGG contribution) and independent variables.

For the execution of these analyses, we utilized Python for the Random Forest algorithm, owing to its robust machine learning libraries, and STATA for conducting the non-parametric tests, parametric tests, and Ordinary Least Squares (OLS) regression.

## 5 Results

In this section, we delineate our research findings, commencing with insights from the qualitative study and transitioning to the outcomes of the lab-in-the-field experiment. The qualitative results shed light on the multifaceted challenges, determinants of cooperation, and anticipated outcomes. Participant quotations, rendered in English, are showcased in Appendix Table 1A for richer context. For confidentiality, these quotes are anonymized. Using the qualitative insights as a bedrock, we subsequently delve into the lab-in-the-field experiment’s results, offering empirical validations and deeper perspectives on the dynamics of interpersonal cooperation amidst the pandemic.

### 5.1 Qualitative study

From the data analysis, a clear distinction arises between the experience of cooperation in the healthcare sector and that in manufacturing. In the healthcare sector, the objective of the workers was to face the emergency, while also resisting the “new normality” imposed by the pandemic. Healthcare workers confronted a specific set of challenges for an extended duration, with interpersonal cooperation playing a crucial role in their work environment. They were tasked with *delivering extraordinary effort*, where extraordinary encompassed both exceptional in terms of quality and quantity of work, and deviation from the established norms. Participants described managing the fear of contagion, coping with worsening working conditions, grappling with a decline in the quality of care, dealing with uncertainty in planning, navigating the confusion of professional roles, adapting to non-standardized decision-making processes, facing emotional burden, and experiencing limitations in their personal lives (#1–8). Certain challenges were particularly severe for cross-border healthcare workers, who found themselves compelled to undertake lengthy journeys between their residences and workplaces or to remain in temporary lodgings in Switzerland (#9–10).

Overcoming these challenges depended on several *individual, institutional, and interpersonal conditions*. Characteristics related to the worker’s personality or professional identity – such as optimism, resilience, trust in their colleagues and in the institution, adaptability, and work ethics – played a crucial role in enabling healthcare providers to cope with the extraordinary workload (#11–15). The leadership provided by direct superiors and the availability of spaces and time for effective communication were instrumental in facilitating effective cooperation, as well as institutional support and timely and consistent information (#16–18). Beyond these individual and institutional conditions, however, the strength of the group was the true driving force behind the extraordinary effort exerted by healthcare providers (#19). Several elements contributed to this cohesion and cooperation within the team: the presence of an external enemy, the virus, created a sense of shared purpose and unity; the high stakes involved, namely the care of others, further reinforced the commitment to collective action; the hospital, as a defined and bounded space and time, provided a significant divide between individuals inside and outside its confines, and reinforced the sense of belonging among those within; the urgent time frame imposed by the waves of the pandemic heightened the need for collaborative efforts; the shared condition of ignorance, with all team members grappling with the uncertainties of the virus, fostered a sense of togetherness and mutual support (#20–25). These factors contributed to the development of a strong collective identity where mutual recognition played an important role: the healthcare workers acknowledged their shared experiences and goals, therefore they perceived themselves as strongly connected to each other and as being part of a functional and interconnected group. This group was characterized by the attenuation of formal differences, the intensification of functional specificities, the transcendence of traditional roles, and the promotion of interprofessional attitudes. In such a context, cooperation could flourish (#26).

However, as time progressed, another main challenge surfaced, related to the *legitimacy* of the extraordinary effort performed by healthcare providers. Participants described how the enthusiasm experienced during the first wave gradually diminished. This decline was not solely due to physical exhaustion but also resulted from a perceived decrease in their sense of purpose and appreciation for their work. The “extraordinary effort,” which once they considered necessary, became unacceptable to them due to the erosion of the principles that had previously justified it. These principles included the exceptional nature of the event due to its unpredictability and transience, the recognition from hierarchies of the extraordinary nature of their work, and the shared commitment of all stakeholders (healthcare providers, patients, and society) in the fight against the common enemy. The participants expressed feelings of being disrespected and unrecognized as the pandemic advanced through its waves: with the pandemic no longer catching them by surprise, their expectation for improved hospital organization heightened; additionally, they bitterly perceived their exceptional efforts as taken for granted; finally, they believed that a significant number of individuals, including both patients and colleagues, were neglecting their responsibilities, rejecting containment measures or vaccination (#27–31). This situation undermined their commitment with patients, hierarchical structures, and colleagues. Consequently, many participants responded by withdrawing and resorting to bureaucratic actions, which reflected a retreat from active cooperation and a diminished sense of purpose (#32).

The experience of cooperation of the workers in the manufacturing sector was significantly different. Right from the outset, their stated goal was to recreate a “new normality” within the ongoing pandemic. Their primary challenge revolved around *adapting to smart working*, which entailed modifying the work environment and communication tools while maintaining the same corporate objectives (#33). In this context, smart working has entailed ambivalence, encompassing both opportunities and challenges. On the one hand, it fostered greater organization and focus, optimizing time management and offering increased flexibility, especially for cross-border workers, who could avoid long travels to reach their workplace (#34–37). On the other hand, it posed several obstacles, including the need for leaders to exercise control, the scarcity of interpersonal communication, the absence of informal interactions, the integration of professional and personal life boundaries, and – of course – the necessity to acquire technological skills (#38–43).

Also in this case, effectively managing these challenges relied primarily on *individual, institutional and interpersonal factors*. Of particular importance were the workers’ adherence to the company’s goals, their aptitude for learning – especially regarding the adoption of new communication tools – and their ability to organize themselves in the absence of a structured environment (#44–45). The inputs provided by the direct supervisor or by the company in general also proved to be important in successfully addressing the challenges of the new working conditions. Leaders were expected to demonstrate supportive and visible guidance, actively engaging with their teams and offering personalized direction and oversight even when working remotely. Regular scheduled online meetings were crucial regarding to this. Expressing concern for employees and their well-being through active listening and tangible gestures, such as providing ergonomic chairs delivered to their homes, helped foster a sense of care and support. Furthermore, organizational consistency ensured that measures and guidelines were clear and coherent across all company levels. This gave employees a sense of stability and trust in decision-making (#46–49). Finally, the quality of pre-existing relational assets, encompassing the significance of the group’s history, a sense of belonging, and the nature of informal relationships that had developed before the pandemic, contributed to a foundation of resilience and mutual reliance. The lack of this relational capital could negatively impact on cooperation. On the contrary, the intensity of interdependence within the team influenced the willingness of individuals to cooperate actively: the stronger the perceived interdependence, the more likely people were to engage in cooperative efforts (#50–51).

Unlike healthcare professionals, workers in the manufacturing sector did not express particular suffering due to the prolonged nature of the pandemic. Their objective was to adapt to the new work conditions by addressing challenges and capitalizing on opportunities. Consequently, they embraced a long-term perspective and utilized the duration of the experience as a chance for better learning how to effectively acclimate to it. Ultimately, they did not question the *legitimacy* of their effort and were even hoping that some of the new working conditions could continue also in the future (#52).

To consolidate our findings from both the healthcare and manufacturing worker samples, we identify [MOU1] key factors operating at various levels that enhanced cooperation. Despite the distinct experiences of cooperation in the two professional settings, our qualitative study highlights the presence of individual enabling elements, institutional conditions, and interpersonal driving factors. Each of these played a critical role in promoting and sustaining the extraordinary cooperation among workers during the COVID-19 pandemic. Individual enabling elements encompassed aspects of personality (such as optimism, trust, and resilience), skills (adaptability, learning ability, and organizational competences), as well as professional identity and ethics. Institutional conditions included consistency in information and organization, the availability of effective communication spaces for teams, and supportive and actively present leadership. Interpersonal driving factors involved the intensity and cohesion of the group, the awareness of its interdependence, and a sense of mutual support during the pandemic. Among these various factors, the significance of internal and external *recognition* [MOU2] emerges as a central theme. In both samples, feeling part of a group of people with shared experiences and goals (internal recognition) considerably reinforced willingness and cooperative attitudes. Likewise, receiving signs of respect and acknowledgement for work accomplished amidst the extraordinary circumstances of the pandemic (external recognition) yielded similar outcomes.

On the contrary, the absence of these two forms or recognition diminished cooperative engagement. The *lack of transience* in the new working conditions also arises as pivotal, albeit in different ways. For healthcare workers subjected to extraordinary stress levels, the duration had a negative impact on cooperation. Conversely, for workers in the manufacturing sector who glimpsed new opportunities, the enduring nature of their situation acted as an incentive for adaptation. The *level of stress*, therefore, also emerges as an influential element capable of modulating the impact of recognition and duration.

### 5.2 Lab-in-the-field experiment

Building upon the valuable insights from the qualitative analysis, our research takes a significant step forward by investigating whether the mesosocial and the microsocial factors identified in the qualitative phase are relevant in shaping cooperation within the Public Good Game (PGG).

**Figure 1:**
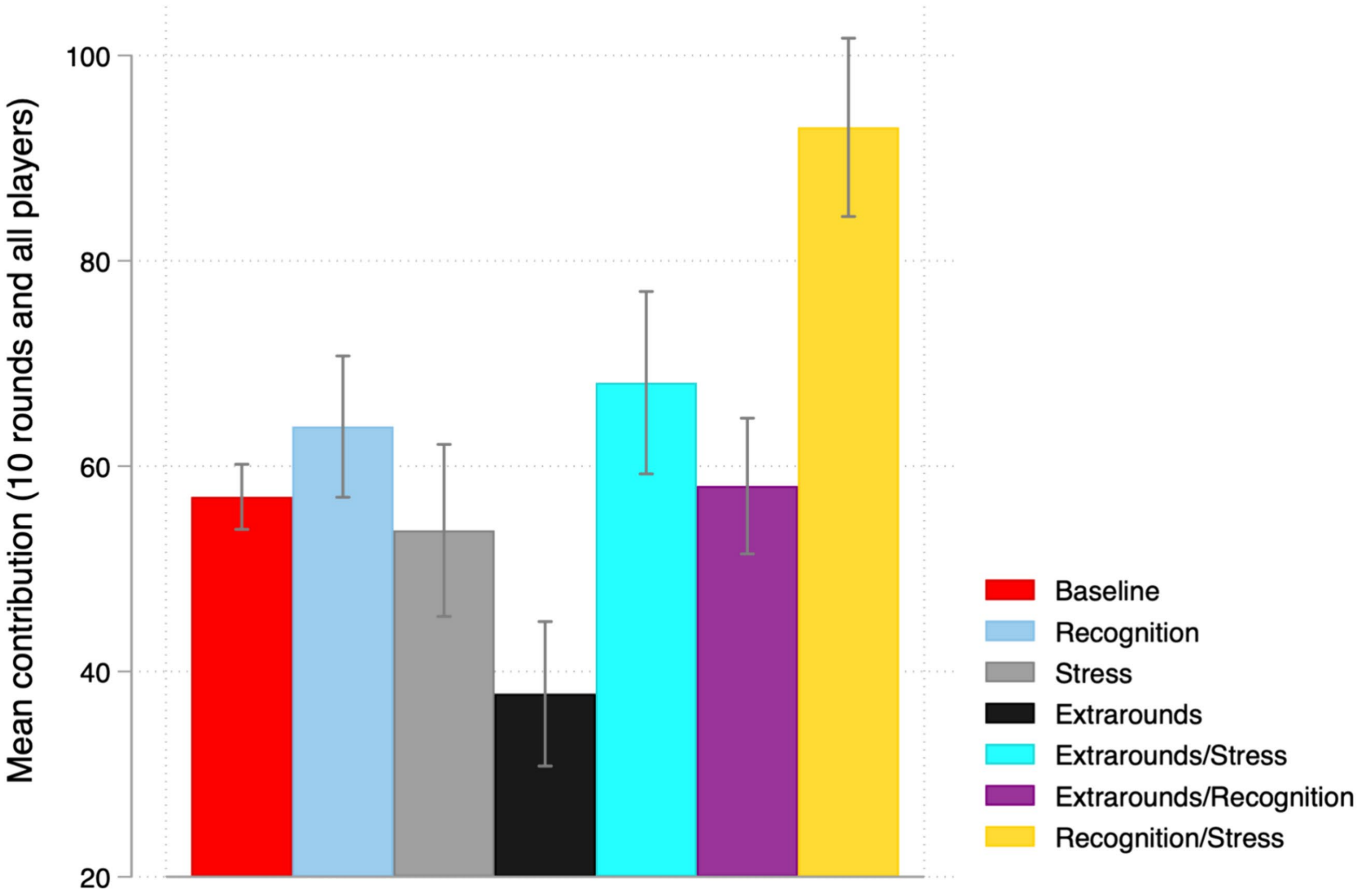
Contributions in the PGG by treatment manipulation and across 10 rounds.

First, we focus on macrosocial conditions. Our objective in this phase is to recreate the circumstances faced by workers during the COVID-19 pandemic in a controlled environment, including the recognition (a lack thereof) of their extraordinary work, the presence (or absence) of time constraints causing stress, and the transient (or non transient) nature of the event. These conditions were implemented as treatment manipulations in our experimental settings, as outlined in Section 3. The results of this exercise are depicted in Table 2 and Figure 1.

**Table 2:** Contributions in the PGG by treatment manipulation and across 10 rounds.

	Average contribution
Baseline	57.0079
Recognition	63.8495
Stress	53.7358
Extrarounds	37.82
Extrarounds/Stress	68.125
Extrarounds/Recognition	58.0583
Recognition/Stress	93

To examine the impact of these treatments on cooperation, we compared the average contributions across all players and the 10 repetitions between the baseline and the main treatment manipulations, which include stress, recognition, and extrarounds. Our analysis reveals significant differences in cooperation levels among these treatments.

Offering recognition to the players results in a marginal increase in cooperation compared to the baseline (*t*-test *p*-value = 0.065, Two-sample Wilcoxon rank-sum (Mann–Whitney) test *p*-value = 0.100). Conversely, the unexpected requirement to play additional rounds significantly hampers cooperation (*t*-test *p* < 0.001, Two-sample Wilcoxon rank-sum (Mann–Whitney) test *p* < 0.001). However, no statistically significant difference in cooperation is found between the baseline and the treatment under stress (*t*-test *p*-value = 0.478, Two-sample Wilcoxon rank-sum (Mann–Whitney) test *p*-value = 0.380), suggesting that the presence of a stressful event *per se* does not increase cooperation.

Further analysis examines the effect of recognition with and without stress. Cooperation is significantly higher in the presence of stress when players receive recognition (*t*-test *p* < 0.001, Two-sample Wilcoxon rank-sum (Mann–Whitney) test *p* < 0.001), suggesting that offering recognition can sustain cooperation during stressful events. Moreover, the difference in cooperation levels between cooperation under stress with and without recognition (Recognition/Stress – Stress) and cooperation levels with and without recognition (Recognition-Baseline) is greater than 0 (32.42), indicating that offering recognition is particularly efficient during stressful situations when players may need an extra boost to sustain cooperation.

Cooperation is also higher in the extraround treatment when there is stress (*t*-test *p* < 0.001, Two-sample Wilcoxon rank-sum (Mann–Whitney) test *p* < 0.001). However, the difference between cooperation levels in the presence of stress with and without extrarounds (Extrarounds/stress – Stress) and cooperation levels with and without extrarounds is not significantly different from 0 (4.7), suggesting that the presence of stress alone cannot help in sustaining cooperation when the transient nature of the event disappears. In contrast, the difference between extrarounds under stress and recognition under stress is highly statistically significant (*t*-test *p* < 0.001, Two-sample Wilcoxon rank-sum (Mann–Whitney) test *p* < 0.001), once again highlighting the importance of properly rewarding cooperation, especially during stressful events, for its sustainability.

**Figure 2:**
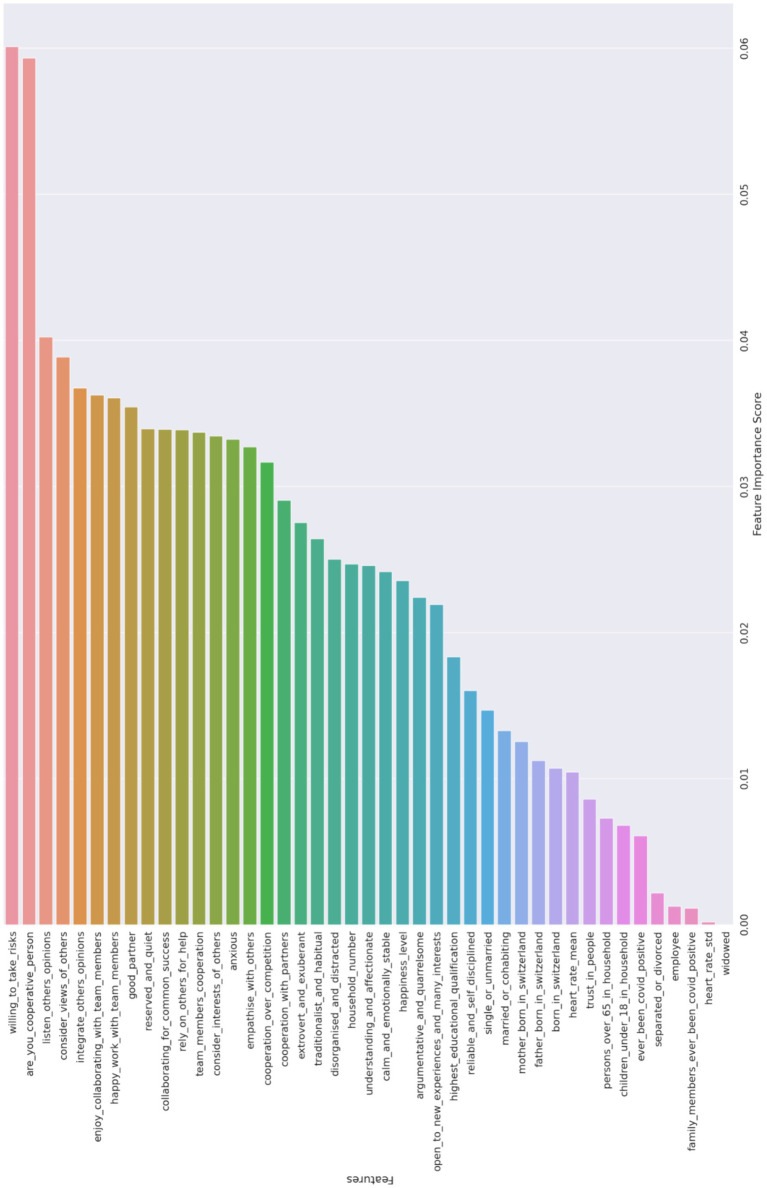
Variables in order of importance for the Random Forest model.

Next, we aim to identify the top variables that influence cooperative behavior across different treatments in the game. The findings are visually presented in Figure 2, representing the results of the Random Forest model described in the methods section. More specifically, Figure 2 is not merely a representation of data; it is a visual synthesis that provides a comprehensive overview of the key variables that emerged as significant influencers of cooperative behavior, as determined by the Random Forest model.

The initial findings depicted in Figure 2 reveal that factors such as group dynamics, social interactions, and personal attributes have a more substantial impact on cooperative behavior than objective situational factors like physiological stress indicators measured through wearable sensors. This emphasizes the significance of personal and social elements over physiological responses in understanding and fostering cooperation. When we closely examine the 10 most important variables selected, a clear picture emerges regarding the specific characteristics and attitudes that strongly influence cooperation within the PGG. These variables encompass a range of factors, including the willingness to take risks, possessing a cooperative personality, actively listening to others’ opinions, considering and integrating diverse viewpoints within a group, and enjoying cooperation with team members. Other important variables are: finding happiness in collective work, the importance of having a dependable partner, displaying a reserved and quiet demeanor, and cultivating a collaborative mindset toward achieving common success. These findings underscore the significance of personal attributes, effective communication skills, and a positive attitude toward teamwork as key drivers of cooperative behavior. Importantly, these results align closely with the insights from the previous qualitative analysis, further bolstering our findings’ validity and reliability.

As depicted in Figure 2, there is a noticeable distinction in the importance of the first two variables compared to the others. This visual observation led us to prioritize these two variables and utilize them as controls in the Ordinary Least Squares (OLS) model.

Table 3 presents the results of the Ordinary Least Squares (OLS) model, as described in the Methods section above. The first three columns focus on the treatments without stress, while the last three columns analyze the treatments with stress. Columns 1 and 4 display the associations between treatments and cooperation while controlling solely for the number of rounds. Columns 2 and 5 introduce additional variables such as age, gender, and being a healthcare worker. Finally, columns 3 and 6 include the two variables selected through the Random Forest Algorithm, namely risk aversion and a self-assessed measure of cooperation.

**Table 3:** OLS contribution at the individual level.

	No stress			Stress		
	(1)	(2)	(3)	(4)	(5)	(6)
	Contribution					
Recognition	6.003 (3.772)	−0.611 (3.670)	−1.600 (3.684)	39.20^***^ (6.598)	45.17^***^ (7.625)	23.57^**^ (7.315)
Extrarounds	−21.39^**^ (6.654)	−8.913 (6.420)	−8.008 (6.430)	11.78 (10.96)	−4.151 (10.83)	−14.37 (9.475)
Rounds	0.133 (0.448)	0.177 (0.416)	0.177 (0.415)	0.255	0.217 (0.759)	0.459 (0.652)
				(0.892)		
25–34		31.11^***^ (7.441)	34.70^***^ (7.572)			
35–44		40.61^***^ (7.560)	44.26^***^ (7.691)		−38.63^***^ (6.556)	−26.43^***^ (6.068)
45–54		19.23^**^ (7.417)	22.26^**^ (7.649)		−43.44 (25.73)	−5.922 (23.31)
Male		−15.19^***^ (2.724)	−15.95^***^ (2.736)		9.060 (25.37)	25.61 (22.78)
Health-care worker		−4.255 (3.213)	−3.795 (3.282)		8.875 (7.404)	61.88^***^ (10.23)
Risk scale			0.151 (0.0875)			−1.097^***^ (0.201)
Cooperative person			0.0471 (0.143)			2.399^***^ (0.367)
Constant	57.14^***^ (2.964)	36.06^***^ (7.786)	18.83 (13.90)	52.40^***^ (6.132)	79.35^***^ (9.130)	−98.04^**^ (31.22)
Observations	634	634	634	123	123	123

Standard errors in parentheses. ^*^p < 0.05, ^**^p < 0.01, ^***^p < 0.001.

The results indicate several significant findings. Firstly, when considering the impact of treatments, offering recognition emerges as a crucial factor in sustaining cooperation under stress. The coefficient for recognition is consistently positive and statistically significant in columns 4–6. Additionally, the absence of transience in the event significantly reduces cooperation when no other control variables are considered. However, when adding controls it becomes insignificant.

Regarding the control variables, men exhibit lower levels of cooperation compared to women in the absence of stress, and this difference is statistically significant. However, in the presence of stress, no significant gender difference is observed. Furthermore, in the presence of stress, risk aversion is found to have a negative relationship with cooperation, indicating that more risk-averse individuals tend to contribute less. Conversely, being a cooperative person, as measured by the self-assessed measure of cooperation, is positively associated with cooperation in the presence of stress. Overall, there is no substantial difference in terms of contribution between healthcare and manufacturing workers as being a healthcare worker shows a positive and statistically significant relationship with contribution only in column 6 of Table 3.

## 6 Discussion and conclusions

This paper delves into the dynamics of interpersonal cooperation in Canton Ticino, Switzerland, during the COVID-19 pandemic. A qualitative study uncovers varied cooperation experiences in the healthcare and manufacturing sectors, elucidating sector-specific challenges and coping strategies. Moreover, our findings underscore the crucial roles of individual, institutional, and interpersonal factors. The lab-in-the-field experiment reinforces the vital role of internal and external recognition, emphasizing that prolonged stress conditions can erode cooperation. The synthesis of qualitative and quantitative methodologies allows a comprehensive exploration of cooperation, particularly within the framework of the Public Good Game (PGG).

From a methodological standpoint, our research offers a pioneering approach in examining cooperation during crises. The qualitative dimension provided a deep dive into the lived experiences during the pandemic, while the behavioral economics experiments offered empirical evidence on cooperation’s underlying mechanisms. The merger of these methodologies illuminated complex patterns, providing a multifaceted research design suitable for other disciplines.

Yet, potential limitations exist. Our study, while offering valuable insights into the dynamics of interpersonal cooperation during the COVID-19 pandemic in Canton Ticino, Switzerland, navigates through inherent methodological and contextual constraints that merit acknowledgment. First and foremost, our reliance on voluntary email responses for participant recruitment may have introduced a selection bias, potentially limiting the representativeness of our sample. This is further complicated by the attrition of healthcare professionals during the recruitment phase, where out of 45 initially interested individuals, 16 were unreachable, raising concerns about the possible impact on the study’s findings due to non-random sample attrition. Moreover, the geographical specificity of our study, focused solely on Canton Ticino, coupled with the concentration on only the healthcare and manufacturing sectors, could restrict the generalizability of our results. While this focus allows for a deep exploration of these sectors during an unprecedented global crisis, it may not capture the full spectrum of cooperation dynamics present in other sectors or regions, which could respond differently to similar stressors. The experimental component of our research, conducted in a lab-in-the-field setting, while innovative, encounters limitations in sample size. The number of participants in these experiments was relatively small, necessitating caution in the extrapolation of results. This constraint underscores the challenge of achieving generalizable findings from experimental data, a common hurdle in behavioral economics research that requires careful consideration in interpreting and applying our results. Furthermore, while the integration of qualitative and quantitative methods enriches our understanding of cooperation, this methodological amalgamation introduces complexities in data synthesis and interpretation. Balancing the depth of qualitative insights with the empirical rigor of quantitative analysis poses challenges, particularly in ensuring that the nuanced, context-specific findings from the qualitative study are adequately reflected in the broader quantitative analysis.

Lastly, the timing of the study, amid the COVID-19 pandemic, presents both an opportunity and a limitation. While it offers a unique lens through which to view cooperation under crisis conditions, it also means that the findings are influenced by the extraordinary circumstances of the pandemic. This context-specific factor may affect the durability of our conclusions and their applicability to other crisis or non-crisis conditions, necessitating further research to explore the persistence of observed behaviors beyond the pandemic context.

However, the insights from this research have critical implications for crisis management. They highlight the pivotal role of recognition in maintaining cooperation during stressful times. The significance of both a strong collective identity and institutional acknowledgment underscores the need for effective collaboration during crises. Looking ahead, future studies should address these constraints, broadening their scope to provide a more holistic perspective on cooperation dynamics in diverse scenarios. This will aid in crafting strategies to foster collective action during global challenges.

## Data availability statement

The raw data supporting the conclusions of this article will be made available by the authors, without undue reservation.

## Ethics statement

The studies involving humans were approved by Comitato etico cantonale c/o Ufficio di sanità Via Orico 5 6501 Bellinzona. The studies were conducted in accordance with the local legislation and institutional requirements. The participants provided their written informed consent to participate in this study.

## Author contributions

VR: Conceptualization, Data curation, Formal analysis, Investigation, Methodology, Software, Supervision, Validation, Visualization, Writing – original draft, Writing – review & editing. MB: Conceptualization, Software, Validation, Writing – review & editing. GL: Conceptualization, Software, Writing – review & editing. ML: Formal analysis, Writing – review & editing. LU: Conceptualization, Resources, Supervision, Validation, Writing – review & editing. SD’O: Formal analysis, Software, Validation, Writing – review & editing. MR: Formal analysis, Visualization, Writing – review & editing. LB: Conceptualization, Validation, Writing – review & editing. MC: Conceptualization, Data curation, Formal analysis, Funding acquisition, Investigation, Methodology, Project administration, Supervision, Validation, Writing – original draft, Writing – review & editing.
