# Supplementary material for: Crucial and fragile: a multi-methods and multi-disciplinary study of cooperation in the aftermath of the COVID-19 pandemic
Source: Front Public Health. 2024 May 16;12:1368056. doi: 10.3389/fpubh.2024.1368056 (PMC11137170; doi:10.3389/fpubh.2024.1368056)
Supplement: Supplementary file 1 [file Data_Sheet_1.docx]

# Appendix

## Table 1A. Exemplary quotes from participants

##

| **Themes** | **Quote** | **Supporting quotes** |
| --- | --- | --- |
| *Healthcare workers* | | |
| Challenges: delivering extraordinary effort | #1 | *We had these sick patients, with a disease that was spreading all over the world, and we thought: who knows if we are protected enough? And when one of us got sick, then we realized that COVID was among us. That was the first moment of fear and disorientation.* |
|  | #2 | *I took part in these grueling shifts during the first wave, shifts lasting 12 to 13 hours under precarious conditions. We were dressed significantly, and all of this led to exhaustion and dehydration. Our primary needs were set aside to keep up with this first wave that crashed over us like a tsunami.* |
|  | #3 | *Normally in intensive care, we make little bows for the patients (…) [During covid] the priorities were different. (…) We had to lower our quality standards. We focused on survival. Our goal was to have the patient alive at the end of the shift, neglecting many things that are normally done.* |
|  | #4 | *This was the mode: from day to night, situations changed, information changed, everything was constantly changing.* |
|  | #5 | *Every day was a new situation and a change. Resetting.* |
|  | #6 | *The responsibility you felt was so great because most of the doctors did not know the respirator and so everything was your responsibility even if you were a nurse. We were taking on responsibilities that were not ours.* |
|  | #7 | *We had to choose which patients to treat. It was demanding to make ethically difficult decisions in such short timeframes, especially for severely ill patients who arrived at the last moment, and we had to quickly decide whether they should undergo intubation and advanced resuscitation measures. This was tough, and it was also tough to communicate our decision to the patients and their families. Because in the end, many times, we had to decide. Our role condemned us to being decisive.* |
|  | #8 | *The first wave I was also working 12, 13 hours a day. I have a child, my husband was at home because there was the 'lockdown', I would come home and if I met my daughter I couldn't go into her bedroom. In all that time, I wasn't there for her.* |
|  | #9 | *It was very hard for cross-borders workers. Both my wife and I used to leave three hours earlier to get to work, stay there ten hours, and come home three hours late (…). I mean, my wife used to get up at four to get to the hospital in Lugano at seven.* |
|  | #10 | *When I was working long shifts, I would stay in a hotel, but since I have two young children at home, I tried to avoid it.* |
| Individual conditions | #11 | *Temperamentally I'm open, sunny, so I probably don't have big problems to adapt in these situations. I don't look at the one who has a sourpuss etc. I'm here to work, we work together. Let's go.* |
|  | #12 | *I wanted to help all these people because I find this to be an absurd injustice. I know I am a person who can help, who can do good.* |
|  | #13 | *I certainly have internal resources. I am very flexible. I have hope, I hope things will go the right way.* |
|  | #14 | *It is part of the ADN of those who work in the emergency: if there is something to be done, it is done. And you don't do it because there is covid. It is done, full stop.* |
|  | #15 | *I never worried about illness because of the duty I had towards work, my colleagues, and my patients. So, I never even had the issue of saying, 'I'll get sick, I won't get sick.' The sense of duty was more important.* |
| Institutional conditions | #16 | *Even the chiefs... a great empathy, a listening, a presence that plays its part.* |
|  | #17 | *Certainly, trust was the basis. In general, thinking about the organization, it was also the fact that the management was there and communicated well daily.* |
|  | #18 | *We talked. We talked a lot. Whatever happened, we shared it. It was a constant search for solutions, ideas, and bringing to light what needed to be done, or what was causing us an issue, so more inputs came in.* |
| Interpersonal conditions | #19 | *This is the only support and the only reason that has kept me going until now: my colleagues, my team.* |
|  | #20 | *We all found ourselves in the same situation. From the head doctor to the last of the colleagues who just started: there is this new problem, and we have all tried in a helpful way to collaborate in the cause.* |
|  | #21 | *We were all focused on fighting the same way, regardless of role. Assistants, head clinicians, nurses, we were all the same.* |
|  | #22 | *In my opinion, it favored being naked and unarmed in the face of something unknown. So, everyone without clothes, all equal. This allowed us to make ourselves all equal. Without prejudices. In that moment, there are no more differences. The only difference concerns the fact that one person knows how to do one thing, and another person knows how to do something else.* |
|  | #23 | *We were all in the same boat, all with the same problems of not seeing children, grandchildren, parents. There was a great sharing of experiences.* |
|  | #24 | *All together, we were aware of an exceptional situation where there's little to complain about, and much to roll up one's sleeves for. So, you eliminate all the peripheral things and get to the substance: here, we must work, together, and save the patients, we must stabilize them and pull them out of this situation. Let’s go and work.* |
|  | #25 | *It is difficult to explain but (…) this spirit of cohesion was something absolutely magnificent.* |
|  | #26 | *The wonderful thing is that you are there in a group of people from different locations, you may have never seen or worked with before, but despite everything, there is help. There is collaboration. It's astonishing. You think that with new people it takes time, and then a group so diverse, with all age groups, all levels of experience... and yet here, there was a paradox! Actually there was excellent collaboration. All obstacles were set aside: we were there to collaborate, to help each other, because we all had to row together in the same direction.* |
| Legitimacy of the effort | #27 | *In the first wave, many gave their all because it was believed to come to an end. After the first wave, we thought: ‘People will understand, everything will start to subside...’. And yet...* |
|  | #28 | *After the first month of the second wave, I sensed a lot of frustration because the expectation was high, saying: 'We've already gone through a first wave, we will surely handle the second one better since...'. Which, on the contrary, didn't happen at all.* |
|  | #29 | *In this second wave - with fewer staff available because other centers have kept their activities - I was asked to have people work 12-hour shifts. I work in this field, I know what it means, I know what it means to work in isolation, wearing the gown, the mask... (…) Arriving at the second wave with the same issues, this is unacceptable.* |
|  | #30 | *Even today, more than a year has gone by [since the start of the pandemic] and even today in some intensive care units the timetable is received every fortnight, or else late, and you never know what to do. Maybe you arrive on the 25th of the month for the first one. (...) A year without planning life outside is hard. They took advantage of us.* |
|  | #31 | *The’ extraordinary’ should last a short time. Instead, it is an extraordinary that is lasting, that is becoming ordinary, and this is devastating.* |
|  | #32 | *[Cooperation] has been hindered, in my opinion, by the non-recognition of the work done, by the lack of feedback, by the non-consideration (...). And this is annoying because you get your hands dirty, you do it for a just cause, you don't claim trophies, not even gratitude. However, acknowledging that we’re all there... The absence of this recognition hampers enthusiasm, which in turn hampers work.* |
| *Manufacturing workers* | | |
| Challenges: adapting to smartworking | #33 | *We continued doing the same things in different ways.* |
|  | #34 | *We are more organized. In the sense that, if you are in an office like ours - we are in an open space divided into two blocks - maybe you often turn around, see your colleagues, have something in mind and interrupt them. Whereas now you know that you can call, and the other person may well not answer but will call you back (...) or you will arrange meetings.* |
|  | #35 | *Distance compels you to engage in perhaps less frequent but more organized exchanges.* |
|  | #36 | *I'm less tired, I work more, I'm much more productive, and then there's also the aspect of silence.* |
|  | #37 | *Through video calls, you enter a more intimate part of a person, and therefore you get to know them even better: dogs barking, children doing their homework...* |
|  | #38 | *You don't see the colleagues and you don't know what they're doing. You can only trust.* |
|  | #39 | *I was antisocial in a social context; so maybe I was breaking out of my antisocial shell. Now, however, I'm antisocial in an antisocial context. We are isolated with few bridges.* |
|  | #40 | *Even now [on Teams] at first you ask, “How are you?". But you don't have the daily routine.* |
|  | #41 | *Seeing faces' is something that is missing. When giving presentations with 40 people, you stand up and see their faces as you speak. With Teams, when you share the screen, you cannot see people's faces and you cannot gauge their reactions. (...) Then you miss a bit the coffee breaks together, the chats....* |
|  | #42 | *You can't build some 'team' spirit by being at a distance, no matter how much every week we have a 'meeting' with everyone present…* |
|  | #43 | *Before, you would wake up, get in your car, have the commute, and enter a place dedicated to work. Now there's no change of context, and you find yourself working 24/7.* |
| Individual conditions | #44 | *Fortunately, I am quite a disciplined person in my own right, I could give myself a structure even if I was at home.* |
|  | #45 | *One no longer worked for personal goals as maybe before, but mainly for company goals. Because if the company went wrong, I was in the way too.* |
| Institutional conditions | #46 | *We were provided support to purchase anything that could be useful for working from home, and it was very beneficial. It's a form of recognition.* |
|  | #47 | *There's the person who was already working autonomously and has become even more independent, so I gave them less attention. There's the person who felt displaced, and I dedicated more time to them. It's important to personalize.* |
|  | #48 | *It's all more based on results rather than control. There's a shift towards empowering individuals and focusing on outcomes.* |
|  | #49 | *I meet with the entire team once a week. I dedicate half an hour to everyone on Monday mornings. Then I meet the managers once a week to align everyone on all the activities. It's a crucial exchange because it allows the team to coordinate internally, even autonomously.* |
| Interpersonal conditions | #50 | *I had already gotten to know them [colleagues], and we often had coffee breaks together. I know a bit about their lives, so even when I call them, there's something to talk about. However, if I were a newcomer who didn't know anything, I would feel very isolated because you can't build some team spirit from a distance.* |
|  | #51 | *Relationships remained strong when there was a strong work connection, but when there wasn't a strong work connection, this informal social aspect broke down a bit.* |
| Legitimacy of the effort | #52 | *I have seen the pros and cons in everything, so in my opinion, the right balance lies somewhere in between. I'd like to stay at home for a while, maybe selecting the days when one has to make a presentation, needs absolute silence and 100 per cent concentration. For the other half of the time, even if I drive three hours a day, I'd like to be together with more people (...). In my opinion it would be just perfect. I hope that as we realize that we also work well at a distance, maybe they will keep it.* |

## 
